# The complete chloroplast genome of *Atractylodes japonica* Koidz. ex Kitam. and its phylogenetic inference

**DOI:** 10.1080/23802359.2021.1927217

**Published:** 2021-06-29

**Authors:** Mengmeng Shi, Hongbo Xie, Chunying Zhao, Linchun Shi, Jinxin Liu, Zhongsi Li

**Affiliations:** a Hebei Key Laboratory of Study and Exploitation of Chinese Medicine, Chengde Medical University, Chengde, China; b Chinese Academy of Medical Sciences and Peking Union Medical College, Institute of Medicinal Plant Development, Beijing, China

**Keywords:** *Atractylodes japonica*, chloroplast genome, phylogenetic relationship, Asteraceae, maximum likelihood

## Abstract

*Atractylodes japonica* Koidz. ex Kitam. is a perennial herbal plant, and its dried rhizomes have been widely used as traditional medicine in China and Japan. In this study, we assembled and annotated the complete chloroplast (cp) genome sequence of *A. japonica* using the high-throughput sequencing approach. The cp genome of *A. japonica* is 153,208 bp in length with the overall GC content of 37.7%, including two inverted repeat (IR) regions of 25,147 bp, which was separated by a large single-copy (LSC) region of 84,255 bp and a small single-copy (SSC) region of 18,659 bp. 113 unique genes were annotated in the genome, including 80 protein-coding genes, 29 represented tRNA genes, and four denoted rRNA genes. A maximum-likelihood phylogenetic analysis with 38 complete cp sequences showed that *Atractylodes* formed a monophyletic clade, and *A. japonica* and *A. koreana* formed a subclade in *Atractylodes*. This study provides the chloroplast genome structure features and phylogenetic relationship of *A. japonica*.

*Atractylodes japonica* Koidz. ex Kitam. (Chinese name ‘Guancangzhu’) is a perennial herbal plant in the *Atractylodes* genus of Asteraceae family, which has potential medicinal value. The crude drug derived from the rhizomes of *A. japonica* has been used as the local medicinal ‘Cangzhu (So-jutsu, in Japanese)’ in Jilin Province of China (Rui and Chou 2020), however, it is not an official resource of ‘Cangzhu’ in Chinese pharmacopoeia for a long time (Commission 2020). In addition, the rhizomes of *A. japonica* have been classified into ‘Baizhu (Byaku-jutsu, in Japanese)’ in the Japanese pharmacopoeia (Kim et al. 2016). Although *A. japonica* has been treated as a synonym of *A. chinensis* according to the Flora of China (Linrong 1987), the mainly pharmacological active ingredients of the crude drugs are different. For instance, the content of atractylodin in *A. chinensis* is higher than *A. japonica* (Cho et al. 2016; Kim et al. 2018; Jeong et al. 2020). The purpose of this study was to analyze the structure of the complete cp genome of *A. japonica* and clarify its phylogenetic relationship with other species in *Atractylodes*.

Fresh leaves of *Atractylodes japonica* were collected from Tonghua City, Jilin Province (N41°43′, E125°56′). Its voucher specimen was deposited in the herbarium at the Chengde Medical University (http://www.cdmc.edu.cn/) with the voucher number as HPAB0017. Total genomic DNA was extracted from the leaf tissue using modified CTAB method (Porebski et al. 1997). The quantity and quality of the DNA were examined using Qubit 4.0 (Thermo Fisher Scientific Inc., USA). Purified DNA was used to construct sequencing library according to the TruSeq DNA PCR-free library preparation guide. The Illumina NovaSeq platform was employed to conduct high-throughput sequencing, and approximately 1.3 GB of raw data was generated with 150 bp paired-end read lengths. The sequencing adapter and low-quality reads were filtered using Trimmonmatic v0.38 (Bolger et al. 2014), and only the ‘paired’ output files were used for subsequent analysis. The complete chloroplast genome was assembled by the organelle assembly NOVOPlasty v4.2.1(Nicolas et al. 2017). The insert size was set to 350 bp and the complete cp genome of *A. lancea* (Accession number: NC_037483.1) was selected as a reference in the configure file of NOVOPlasty. The CPGAVAS2 (www.herbalgenomics.org/cpgavas2) (Shi et al. 2019) was used to annotate protein-coding, rRNA, and tRNA genes of cp genome and visualization with the default parameters. The final chloroplast complete genome of *A. japonica* was submitted to GenBank (Accession number: MW301112).

The chloroplast genome sequence of *Atractylodes japonica* was 153,208 bp in length and showed a conserved quadripartite structure like most angiosperm plants such as *Withania somnifera* (Mehmood et al. 2020) and *Magnolia polytepala* (Sun et al. 2020), with two reverse repeated regions (IRa and IRb) of 25,147 bp in length. The repeat regions divided the entire genome into two single-copy regions, namely a small single-copy region (SSC) and a large single-copy region (LSC) with 18,659 bp and 84,255 bp, respectively. The total GC content of the chloroplast genome was 37.7%. 113 unique genes were annotated in the genome, including 80 protein-coding genes, 29 represented tRNA genes, and four denoted rRNA genes (*rrn23S*, *rrn16S*, *rrn5S*, and *rrn4.5S*). Furthermore, 17 genes were annotated as containing introns, 10 (seven protein-coding and three tRNA genes) of which contained one intron and seven of which (*rps12*, *ycf3*, *ndhB*, *rpl2*, *clpP*, *trnA-UGC* and *trnI-GAU*) contained two introns. Moreover, *petB*, *petD*, and *rpl16* have small exons, and the length of these small exons for the three genes are 6 bp, 8 bp and 9 bp, respectively. Finally *rps12* was identified as a trans-splicing gene. In addition, there is another complete chloroplast genome sequence (Accession number: MT834523) of *Atractylodes japonica* can be found in Genbank submitted by Wang et al. (2021), and there are lots of annotation errors in this released sequence. Four genes (*psbL*, *ycf15*, *trnV-UAC* and *trnfM-CAU*) failed to be annotated, three genes named *atpB*, *ndhD* and *rpoC2* have been incorrectly annotated with wrong start or stop positions, 17 single nucleotide polymorphism (SNP) sites have been found in 13 protein-coding genes including *atpA*, *atpF*, *ccsA*, *clpP*, *matK*, *ndhA*, *petB*, *psaB*, *rpl20*, *rpoB*, *rpoC1*, *rps19* and *ycf2* comparing to the sequence reported in this study. These annotation errors and SNPs can be found in Supplementary Table 1.

To confirm the phylogenetic location of *A. japonica* in the genus *Atractylodes*, a total of 38 complete chloroplast genomes belong to 9 genera of the Asteraceae family were used for phylogenetic analysis (Cai et al. 2020) based on the Maximum Likelihood (ML) method using RAxML v8.2.12 (Stamatakis 2014) with 1000 bootstrap replicates. *Gerbera jamesonii* and *Ixeris polycephala* were used as the outgroups for tree rooting (Susanna et al. 1995). The phylogenetic tree showed that *Atractylodes* formed a monophyletic clade with a bootstrap value of 100 (Figure 1). *A. macrocephala* formed a branch independently with a bootstrap value of 92*. A. chinensis* and *A. lancea* formed a subclade in *Atractylodes* with a bootstrap value of 99, whereas *A. japonica* and *A. koreana* formed another subclade with a bootstrap value of 89. This study reports the chloroplast structure features of *A. japonica,* which provides valuable genetic information for its phylogenetic location in *Atractylodes* genus.

**Figure 1.**
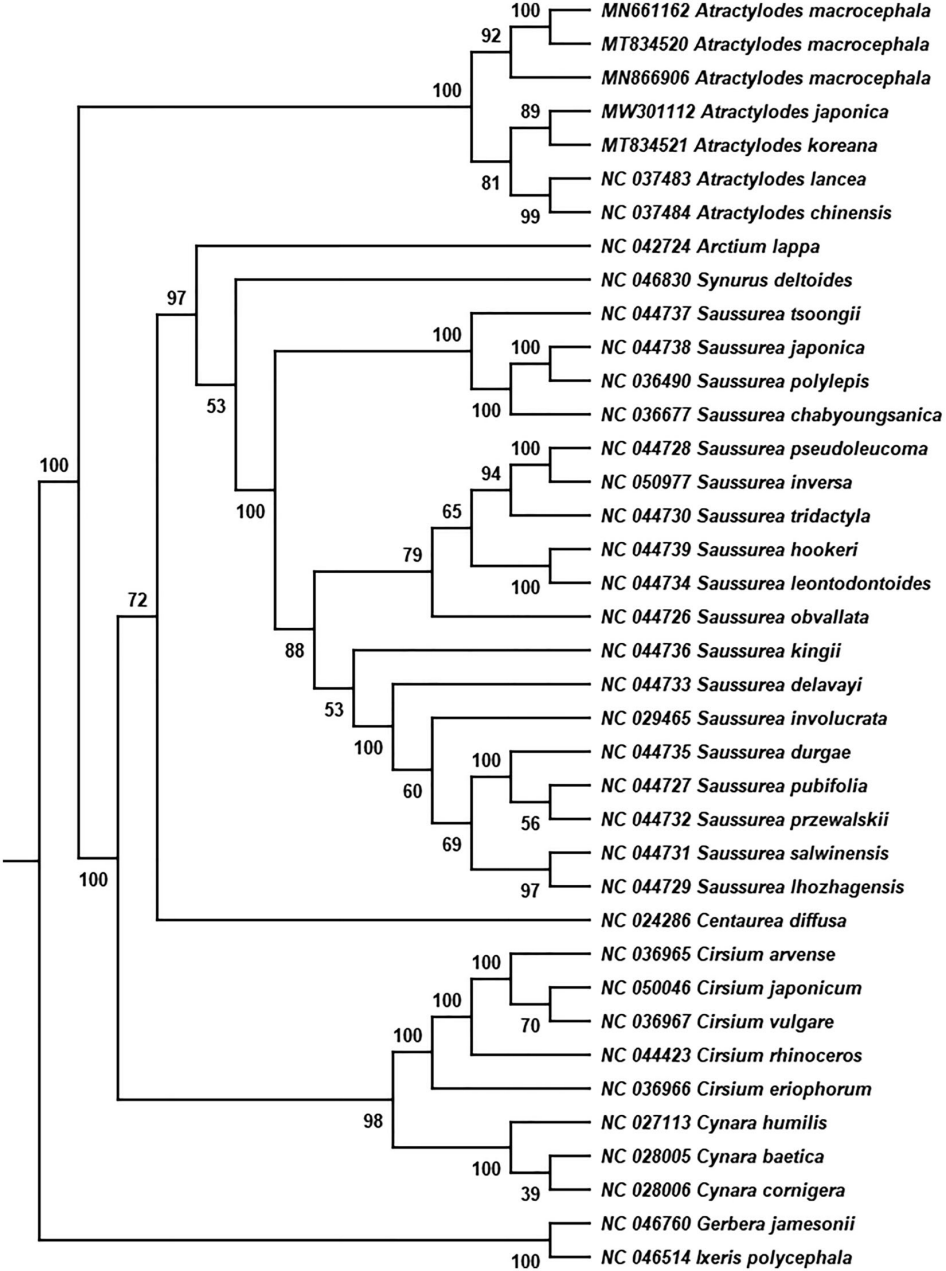
Phylogenetic relationships of *Atractylodes japonica* and additional 37 complete chloroplast genomes. Bootstrap support values are given on the branches.

## Data Availability

The genome sequence data that support the findings of this study are openly available in GenBank of NCBI (https://www.ncbi.nlm.nih.gov/) under the accession no. MW301112. The associated BioProject, SRA, and BioSample numbers are PRJNA682118, SRR13181860, and SAMN16981139 respectively.
